# Self-Assembled Daunorubicin/Epigallocatechin Gallate Nanocomplex for Synergistic Reversal of Chemoresistance in Leukemia

**DOI:** 10.3390/ijms24010381

**Published:** 2022-12-26

**Authors:** Ki Hyun Bae, Fritz Lai, Betul Oruc, Motomi Osato, Qingfeng Chen, Motoichi Kurisawa

**Affiliations:** 1 Institute of Bioengineering and Bioimaging, 31 Biopolis Way, The Nanos, Singapore 138669, Singapore; 2 Institute of Molecular and Cell Biology, 61 Biopolis Drive, The Proteos, Singapore 138673, Singapore; 3 Cancer Science Institute of Singapore, National University of Singapore, 14 Medical Drive, Singapore 117599, Singapore; 4 Graduate School of Advanced Science and Technology, Japan Advanced Institute of Science and Technology, 1-1 Asahidai, Nomi 923-1292, Ishikawa, Japan

**Keywords:** daunorubicin, epigallocatechin gallate, nanocomplex, chemoresistance, leukemia

## Abstract

Chemoresistance is one of the major challenges for the treatment of acute myeloid leukemia. Epigallocatechin gallate (EGCG), a bioactive polyphenol from green tea, has attracted immense interest as a potential chemosensitizer, but its application is limited due to the need for effective formulations capable of co-delivering EGCG and anti-leukemic drugs. Herein, we describe the formation and characterization of a micellar nanocomplex self-assembled from EGCG and daunorubicin, an anthracycline drug for the first-line treatment of acute myeloid leukemia. This nanocomplex was highly stable at pH 7.4 but stimulated to release the incorporated daunorubicin at pH 5.5, mimicking an acidic endosomal environment. More importantly, the nanocomplex exhibited superior cytotoxic efficacy against multidrug-resistant human leukemia cells over free daunorubicin by achieving a strong synergism, as supported by median-effect plot analysis. The observed chemosensitizing effect was in association with enhanced nucleus accumulation of daunorubicin, elevation of intracellular reactive oxygen species and caspase-mediated apoptosis induction. Our study presents a promising strategy for circumventing chemoresistance for more effective leukemia therapy.

## 1. Introduction

Acute myeloid leukemia (AML) is the most common leukemia among the adult population, accounting for an estimated 1,000,000 new cases and 147,100 deaths annually in the world [1]. The first-line treatment of AML primarily involves intensive chemotherapy using daunorubicin (DNR) and other anthracycline drugs, such as doxorubicin, idarubicin and mitoxantrone [2]. Although complete remission is achieved in nearly 80% of patients, leukemic blasts tend to develop resistance to anthracycline treatment over time, contributing to disease relapse and poor long-term outcomes [3]. The treatment of resistant leukemic blasts requires higher doses of anthracycline drugs or longer durations of treatment, but this approach inevitably increases the risk of deleterious side effects, such as cardiotoxicity, hepatotoxicity, neuropathy and myelosuppression [4]. In spite of the discovery of various anti-leukemic drugs, the relapse rates still remain high at 40% in patients younger than 60 years and 10–20% of patients above 60 years, driving the demand for alternative therapeutic strategies [5].

Epigallocatechin gallate (EGCG) is the major polyphenolic compound present in green tea and has been regarded to have diverse health benefits, including anticancer, anti-obesity, neuroprotective and immune-regulatory effects [6,7,8]. EGCG has drawn significant attention for AML treatment because it can inhibit the proliferation of leukemic blasts more preferentially than normal cells [9,10]. Multiple studies have reported the potential of EGCG to overcome chemoresistance by promoting the intracellular accumulation of anthracycline drugs via down-regulation of drug efflux transporters [11,12,13]. Furthermore, EGCG has been documented to enhance drug susceptibility of leukemic cells by generating reactive oxygen species (ROS), such as hydroxyl radical (•OH) and hydrogen peroxide (H_2_O_2_), which can trigger oxidative-stress-mediated cell death [14,15,16]. 

**Figure 1:**
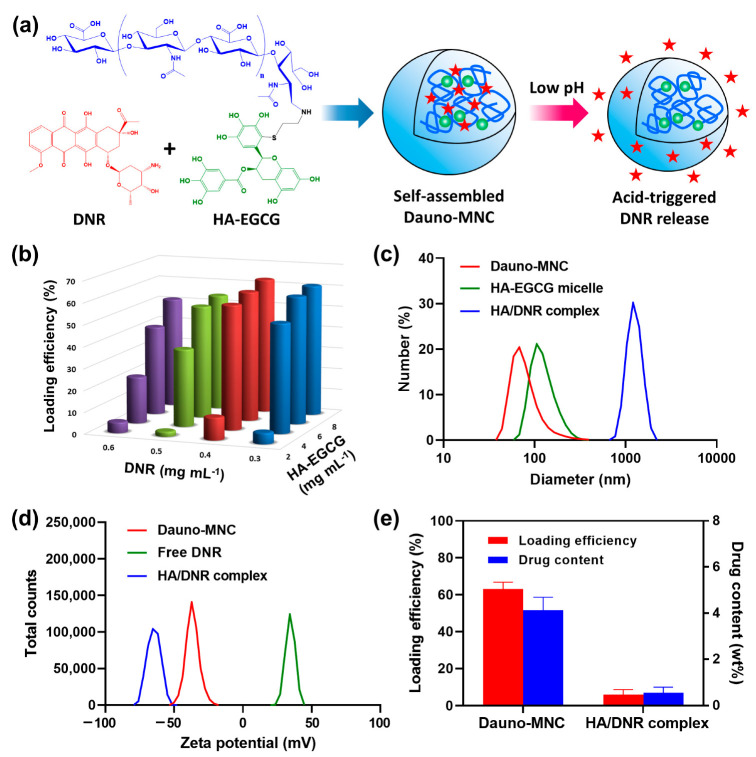
(**a**) Scheme for the formation of Dauno-MNC via self-assembly of DNR and HA–EGCG conjugate and acid-triggered DNR release at low pH. (**b**) Drug-loading efficiency of Dauno-MNC prepared at varying final concentrations of DNR and HA–EGCG conjugate. Data are reported as mean values (*n* = 3). (**c**) Particle-size distribution of Dauno-MNC, HA–EGCG micelle and HA/DNR complex. (**d**) Zeta-potential profiles of Dauno-MNC, free DNR and HA/DNR complex. (**e**) Comparison of loading efficiency and drug content between Dauno-MNC and HA/DNR complex. Mean ± SD (*n* = 3).

We recently developed a well-defined molecular conjugate of hyaluronic acid (HA) and EGCG through the covalent attachment of EGCG to thiol-terminated HA under mild basic conditions [17]. HA is a biodegradable and biocompatible polysaccharide ubiquitous in the human body and has long been used for various biomedical applications, such as drug delivery, tissue engineering and implant coating [18,19,20]. In the present study, we hypothesized that a HA–EGCG conjugate and DNR would undergo self-assembly into a micellar nanocomplex via intermolecular attraction between the DNR and EGCG moiety (Figure 1a). Particularly, multivalent hydrogen-bonding interactions were expected to occur due to the presence of multiple hydrogen bond donors and acceptors on DNR and EGCG molecules [21,22]. The resultant ‘Daunorubicin-loaded Micellar NanoComplex’ (Dauno-MNC) was designed to remain stable at neutral pH but rapidly release the encapsulated DNR in response to acidic pH inside endosomes, thus, improving its nucleus uptake and cytotoxic effect. In the present proof-of-concept study, Dauno-MNC exhibited superior synergistic cytotoxicity, nuclear targeting, intracellular ROS production and caspase-3/7 activity over free DNR in multidrug-resistant HL-60/MX2 cells, demonstrating its potential to reverse chemoresistance for more effective AML therapy. 

## 2. Results and Discussion

### 2.1. Production and Characterization of Dauno-MNC

Dauno-MNC was produced by inducing co-assembly of DNR and HA–EGCG conjugate in aqueous solution and then retrieved using centrifugal filters with an *M*_w_ cutoff of 50 kDa. Since the HCl salt form of DNR is readily ionized and water-soluble, the self-assembly process did not involve the use of any hazardous organic solvents. The centrifugal filtration step allowed for efficient removal of uncomplexed DNR (527.5 Da) and HA–EGCG conjugate (~20.5 kDa) from the mixture, which are much smaller than the *M*_w_ cutoff. The formation of Dauno-MNC was examined under various concentrations of the DNR and HA–EGCG conjugate. As shown in Figure 1b, drug loading efficiency of Dauno-MNC gradually escalated with increasing HA–EGCG concentration, suggesting that higher HA–EGCG concentrations resulted in stronger attractive intermolecular interactions. The drug loading efficiency increased when DNR concentration was raised from 0.3 to 0.4 mg mL^−1^, but began to decrease above the concentration, probably due to the addition of DNR beyond the loading capacity of the HA–EGCG moiety. The maximum loading efficiency (ca. 63%) was achieved when HA–EGCG and DNR concentration was 8 and 0.4 mg mL^−1^, respectively, and the corresponding Dauno-MNC formulation was selected for further investigations. For comparison, HA/DNR complex was produced under the same condition, except the use of unmodified HA instead of HA–EGCG conjugate. 

As depicted in Figure 1c, Dauno-MNC had a far smaller particle size (~68 nm) than that of the HA/DNR complex (~1209 nm). The large particle size of the HA/DNR complex can be explained as a result of uncontrolled aggregation via electrostatic interactions between cationic DNR and anionic HA [23]. While Dauno-MNC exhibited a transparent red solution, the HA/DNR complex appeared opaque, possibly due to the presence of large micron-sized aggregates (Figure S1). Interestingly, Dauno-MNC was found to be smaller than the micelle purely composed of the HA–EGCG conjugate (~106 nm), suggesting that the intermolecular attraction between DNR and EGCG led to the formation of a more densely packed micellar structure. Zeta-potential analysis (Figure 1d) revealed that free DNR had a positively charged structure (+34.2 mV), whereas a negatively charged surface was observed from the Dauno-MNC (−37.3 mV) and HA/DNR complex (−65.6 mV). Since the negative zeta potential of Dauno-MNC and HA/DNR complex was likely caused by the presence of anionic HA molecules on the particle surface, it was conceivable that DNR was fully encapsulated in their interior. It is worth noting that the drug-loading capacity of Dauno-MNC was markedly larger than the HA/DNR complex (Figure 1e). For instance, Dauno-MNC showed approximately 10.6-fold and 7.5-fold enhancements in the loading efficiency and drug content, respectively, compared to the HA/DNR complex. Since DNR and EGCG possess multiple hydrogen bond donors and acceptors, the self-assembly of Dauno-MNC was likely governed by multivalent hydrogen-bonding interactions between the DNR and EGCG moiety [21,22]. Hence, these results suggest that the intermolecular hydrogen bonding promoted more efficient nanoencapsulation of DNR than the electrostatic interactions, leading to the formation of a compact nano-sized MNC structure with improved drug loading. 

### 2.2. Assessment of the Particle Stability of Dauno-MNC 

**Figure 2:**
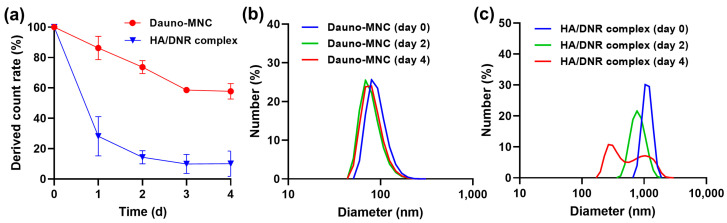
(**a**) Time-course change in derived count rate of Dauno-MNC and HA/DNR complex in PBS at 37 °C. Mean ± SD (*n* = 3). (**b**,**c**) Size-distribution profiles of (**b**) Dauno-MNC and (**c**) HA/DNR complex in PBS measured over 4 days.

Dynamic light-scattering analysis was carried out to examine the stability of the Dauno-MNC and HA/DNR complex in phosphate-buffered saline (PBS) under a physiological environment (pH 7.4 and 37 °C) over 4 days. Specifically, we monitored time-course change in the derived count rate, which is an absolute light-scattering intensity and considered an indicator of nanoparticle integrity [24]. As presented in Figure 2a, the derived count rate of the HA/DNR complex was drastically dropped to ~14% after incubation in PBS for 2 days, indicating the complex was readily dissociated in the physiological condition. In contrast, Dauno-MNC exhibited a much slower decline in the derived count rate (~74% for 2 days) than the HA/DNR complex, reflecting the superior stability of Dauno-MNC. 

This finding was further corroborated by particle-size distribution analysis. There was no significant change in the particle size of Dauno-MNC in PBS over 4 days, indicative of its high stability under the physiological environment (Figure 2b). On the other hand, the HA/DNR complex showed a continuous shift in its particle-size distribution over 4 days in PBS, with the formation of a bimodal mixture peaking at 267.2 nm and 1039 nm on day 4 (Figure 2c). The observed instability in the HA/DNR complex was probably caused by the disruption in the electrostatic interactions between HA and DNR due to the presence of counter ions in PBS (e.g., Na^+^, K^+^, Cl^−^, HPO_4_^2−^). Collectively, the above results demonstrated that the structure of Dauno-MNC was more stable in the physiological condition than the HA/DNR complex. 

### 2.3. Fluorescence Spectroscopy Analysis

**Figure 3:**
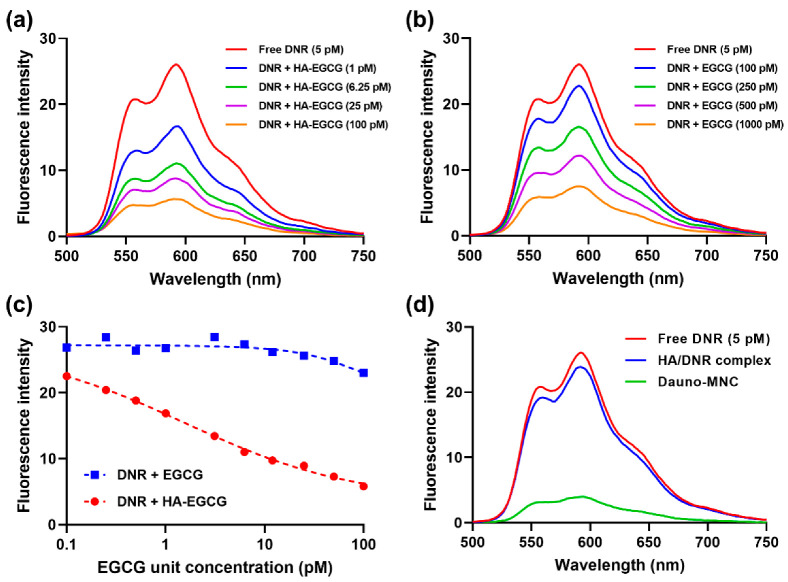
(**a**,**b**) Fluorescence emission spectra (λ_ex_ = 480 nm) of DNR solution (5 pM) mixed with various concentrations of (**a**) HA–EGCG or (**b**) native EGCG. (**c**) Comparison of the fluorescence quenching effect of HA–EGCG and EGCG (λ_em_ = 592 nm). (**d**) Fluorescence emission spectra of Dauno-MNC, HA/DNR complex and free DNR at an equal concentration (5 pM).

The intermolecular binding between the DNR and HA–EGCG conjugate was investigated via fluorescence spectroscopy. As shown in Figure 3a, the free-DNR solution exhibited a characteristic fluorescence emission peak at 592 nm, consistent with the literature [25]. The intrinsic fluorescence of DNR was greatly decreased in a dose-dependent manner upon addition of the HA–EGCG conjugate, indicating the occurrence of intermolecular attraction [21]. In contrast, almost no quenching of DNR fluorescence was observed upon addition of unmodified HA, implying that the EGCG moiety plays an important role in mediating the hydrogen-bonding interactions (Figure S2). 

Notably, the fluorescence-quenching effect of native EGCG was not as strong as that of the HA–EGCG conjugate (Figure 3b). For example, 1000 µM of native EGCG was required to quench DNR fluorescence down to a level achieved by 100 µM of the HA–EGCG conjugate. Quantification of fluorescence intensity (λ_em_ = 592 nm) revealed that the HA–EGCG conjugate was much more effective in binding to DNR than native EGCG at equivalent concentrations (Figure 3c). It has been reported that the HA–EGCG conjugate spontaneously self-assembles into micellar nanoparticles having closely packed EGCG moieties, which can provide a favorable environment for the encapsulation of drug payloads via multiple non-covalent interactions [17,26]. Hence, it was conceivable that the proximal packing of EGCG moieties might contribute to the enhanced DNR-binding affinity of the HA–EGCG conjugate. 

To ascertain whether the fluorescence quenching also occurred within micellar configuration, we compared the fluorescence emission of Dauno-MNC and free DNR at an equal concentration (5 pM). As depicted in Figure 3d, DNR fluorescence was substantially quenched within Dauno-MNC, suggesting that the MNC structure was predominantly maintained by the intermolecular binding between DNR and EGCG moiety. On the other hand, DNR fluorescence was only slightly reduced within the HA/DNR complex, verifying that DNR molecules were not closely packed in the structure of the HA/DNR complex. 

### 2.4. Drug Release Study

**Figure 4:**
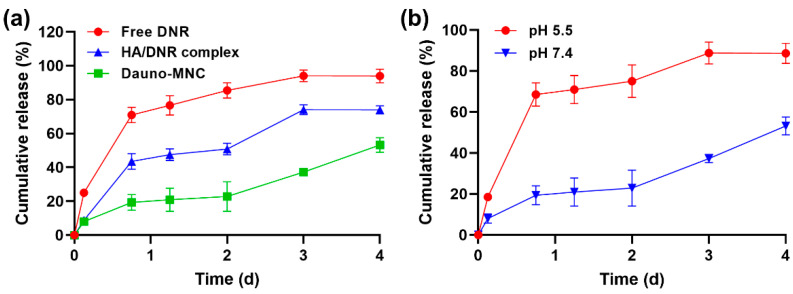
(**a**) Cumulative drug release profiles of Dauno-MNC, HA/DNR complex and free DNR in 10 mM PBS (pH 7.4) over 4 days. (**b**) Cumulative drug release profiles of Dauno-MNC at pH 5.5 and 7.4. Mean ± SD (*n* = 3).

To evaluate the drug release behavior, free-DNR solution, HA/DNR complex and Dauno-MNC having the same amount of DNR (20 µg) were placed in dialysis tubes immersed in 5 mL of 10 mM PBS (pH 7.4). As shown in Figure 4a, free DNR was rapidly diffused out from the dialysis tubes, reaching a cumulative release of 71% and 94% after 18 h and 96 h, respectively. The slower drug release of the HA/DNR complex was probably caused by the delayed diffusion of DNR by electrostatic interactions between cationic DNR and anionic HA. Interestingly, Dauno-MNC exhibited a more sustained drug release profile than the HA/DNR complex. For instance, only about 19% of the drug content was released from Dauno-MNC for 18 h, whereas over 45% was liberated from the HA/DNR complex during the same period of time. The multiple hydrogen-bonding interactions between the DNR and EGCG moiety could be responsible for the sustained drug release behavior of Dauno-MNC. This characteristic is desirable because it can minimize a premature drug leakage during blood circulation before reaching the target cells [27]. 

It is well documented that extracellular fluid pH remains constant at approximately 7.4, whereas the mature endosome has a more acidic environment (pH ~5.5) [28]. Since the cellular site of action of DNR is mainly nucleus [29], a pH-responsive drug release property would offer an effective strategy to improve the therapeutic efficacy of DNR by facilitating its endosomal escape and nucleus targeting [30,31]. Notably, the release of DNR from Dauno-MNC was dramatically accelerated at pH 5.5 when compared to pH 7.4 (Figure 4b). The increased protonation of NH_2_ groups on DNR might be responsible for its faster release from Dauno-MNC at lower pH [32,33]. This pH-sensitive release behavior would be advantageous because it allows for efficient cytosolic delivery and nucleus accumulation of DNR, thereby leading to an improvement in its cytotoxic activity [34]. 

### 2.5. Cytotoxic Efficacy on Multidrug-Resistant AML Cells 

**Figure 5:**
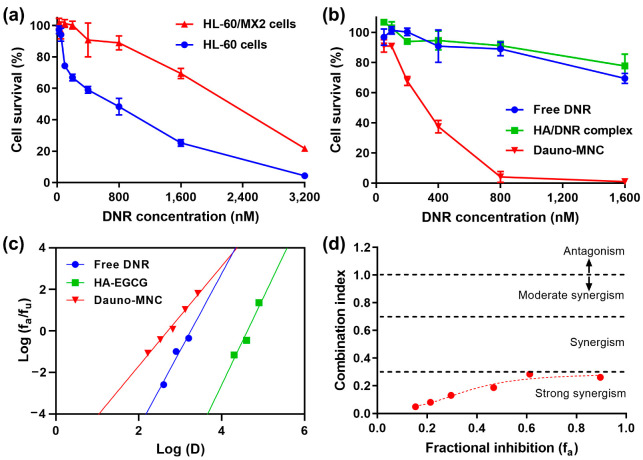
(**a**) Viability of HL-60 and HL-60/MX2 cells treated with free DNR at varying concentrations. (**b**) Cytotoxicity of Dauno-MNC, HA/DNR complex and free DNR against HL-60/MX2 cells. Mean ± SD (*n* = 4). (**c**) Median-effect plots showing eradication of HL-60/MX2 cells treated with free DNR, HA–EGCG or their combination (Dauno-MNC). (**d**) Combination index plot.

In this study, we selected the HL-60/MX2 cell line to investigate the effect of Dauno-MNC on multidrug-resistant AML cells. HL-60/MX2 is a multidrug-resistant variant of the human promyelocytic leukemia HL-60 cell line and has been reported to have cross-resistance to various antileukemic drugs, including DNR, doxorubicin, etoposide, teniposide and bisantrene [35]. As depicted in Figure 5a, HL-60/MX2 cells were more resistant to DNR treatment than the parental HL-60 cells. The IC_50_ value of free DNR was calculated as 2312 and 644 nM for HL-60/MX2 and HL-60 cells, respectively, justifying the use of HL-60/MX2 cells as a representative drug-resistant AML model. Impressively, Dauno-MNC was highly potent in killing HL-60/MX2 cells with a markedly lower IC_50_ value of 316 nM, demonstrating its chemosensitizing ability (Figure 5b). For instance, more than 96% of the cells were eradicated by 800 nM of Dauno-MNC, whereas only a marginal cytotoxicity (~11%) was achieved by the same dose of free DNR. On the other hand, the HA/DNR complex failed to overcome the chemoresistance in HL-60/MX2 cells, as evident from the cytotoxicity profile comparable with free DNR. Of note, the anti-leukemic effect of Dauno-MNC was far greater than that of the HA–EGCG conjugate and native EGCG at an equivalent dose, implying a significant synergistic effect of DNR in combination with the EGCG moiety (Figure S3). 

Next, we conducted median-effect plot analysis to assess the level of synergy of the Dauno-MNC formulation. Cytotoxicity data were first plotted using the linearized median-effect equation [36]. As shown in Figure 5c, the resultant median-effect plots were well fitted to a linear regression model with R-squared values of >0.99. Based on the mutually exclusive model, the combination index (CI) values were calculated at various levels of fractional inhibition. Impressively, all the CI values of Dauno-MNC were smaller than 0.3, indicating that strong synergism occurred between the DNR and HA–EGCG conjugate (Figure 5d). Therefore, it was conceivable that co-delivery of the DNR and HA–EGCG conjugate using Dauno-MNC counteracted the chemoresistance of HL-60/MX2 cells in a synergistic manner. This strong synergy would be advantageous because the dose of DNR needed to acquire the desired cytotoxic effects can be greatly decreased, as evidenced by Figure 5b. Moreover, it can also help in minimizing the risk of harmful side effects associated with high-dose DNR treatment [4]. 

### 2.6. Cellular Uptake and Nucleus Localization of Dauno-MNC 

**Figure 6:**
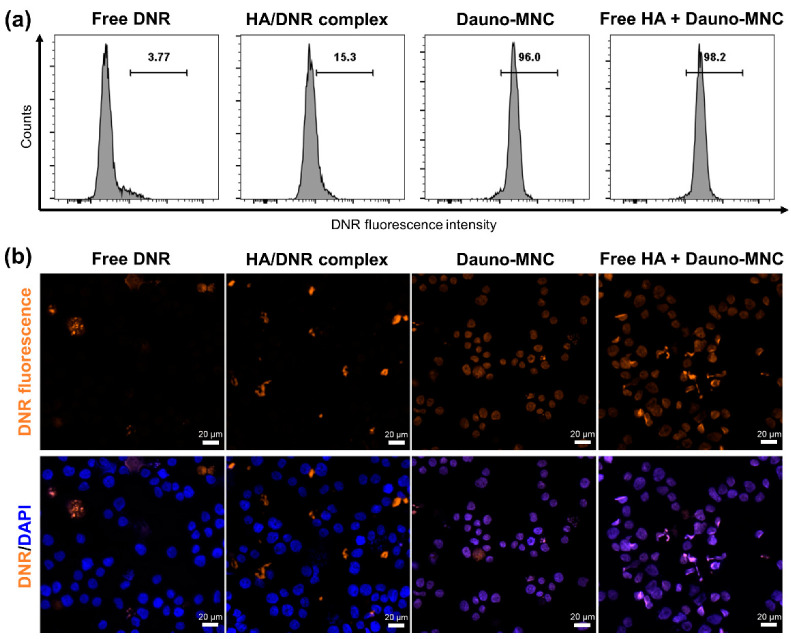
(**a**) Flow cytometric analysis of DNR fluorescence intensity of HL-60/MX2 cells treated for 4 h with free DNR, HA/DNR complex and Dauno-MNC without or with excess free HA at a final DNR concentration of 1.6 µM. The gate was selected after analyzing untreated HL-60/MX2 cells. (**b**) Fluorescence microscopic images of HL-60/MX2 cells subjected to different treatments. Orange and blue fluorescence show the intracellular distribution of DNR and nucleus, respectively. Scale bar = 20 μm.

We reasoned that the observed chemosensitizing effect of Dauno-MNC might be related to the enhancement in cellular uptake because multidrug resistance of leukemia cells is commonly mediated by the decreased drug uptake via overexpression of multiple drug efflux transporters [37]. To answer this question, we conducted flow cytometry analysis to compare the cellular uptake of free DNR, HA/DNR complex and Dauno-MNC at an equivalent DNR dose (1.6 µM). As presented in Figure 6a, only 3.77% of HL-60/MX2 cells was found to have DNR fluorescence after incubation with free DNR for 4 h, suggesting that DNR was highly susceptible to the drug efflux mechanism of the cells. While only a modest increase in DNR uptake was observed from the HA/DNR complex (15.3%), Dauno-MNC promoted a dramatic increase in the cellular uptake to 96%. These results revealed that the chemosensitizing effect of Dauno-MNC was likely attributed to its ability to facilitate the intracellular transport of DNR. Interestingly, the addition of excess free HA as a CD44 blocker had little influence on the internalization of Dauno-MNC (98.2%). Unlike the parental HL-60 cells, which expressed high levels of CD44, almost no expression of CD44 was detected in HL-60/MX2 cells (Figure S4). Hence, this finding suggests that the cellular entry of Dauno-MNC occurred through a non-CD44-dependent pathway. 

Since the mechanism of action of DNR is associated with its intercalation into double-stranded DNA [29], an adequate nucleus localization is a prerequisite for the drug to exert its therapeutic effects. Accordingly, fluorescence microscopy was performed to investigate the nucleus accumulation of free DNR, HA/DNR complex and Dauno-MNC. DAPI was used to stain cell nuclei due to its ability to fluoresce upon binding to double-stranded DNA [38]. As shown in Figure 6b, the fluorescence signal of the free DNR and HA/DNR complex (orange region) did not overlap with that of cell nuclei (blue region). In contrast, the majority of HL-60/MX2 cells treated with Dauno-MNC emitted violet fluorescence signals throughout the nucleus, indicating co-localization of DNR and DNA signals. This co-localization pattern was not affected by the presence of excess free HA, confirming no involvement of CD44 in the nucleus targeting of Dauno-MNC. Taken together, the above results demonstrated that Dauno-MNC enhanced the intracellular uptake and nuclear accumulation of DNR by evading the drug efflux mechanism of HL-60/MX2 cells. 

### 2.7. Intracellular ROS Production and Caspase Activation 

**Figure 7:**
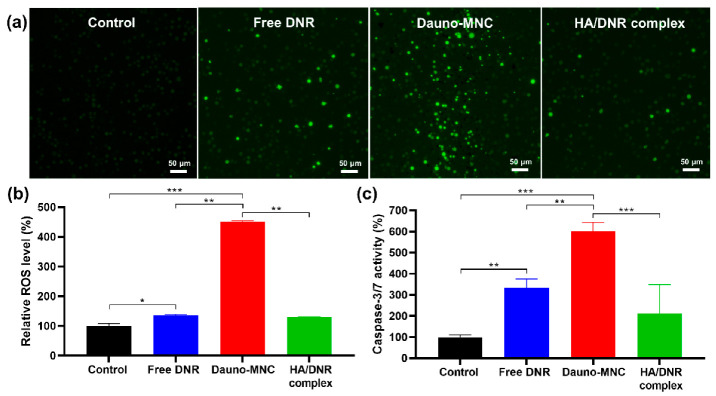
(**a**) Representative CM-H_2_DCFDA staining images of HL-60/MX2 cells treated for 1 h with Dauno-MNC, HA/DNR complex and free DNR at a final DNR concentration of 1.6 µM. ROS-generating cells showed green fluorescence. Scale bar = 50 μm. (**b**) Relative ROS levels of HL-60/MX2 cells that received the same treatment as those in (**a**). (**c**) The effect of different treatments on the caspase-3/7 activity of HL-60/MX2 cells after 1 day of incubation. Mean ± SD (*n* = 6); * *p* < 0.05; ** *p* < 0.01; *** *p* < 0.001.

In addition to drug efflux transporters, augmented antioxidant defense has been recognized as another key contributor to the mechanism of multidrug resistance in leukemic cells [39,40]. Upon cellular internalization, DNR is known to undergo metabolic reduction to generate ROS, such as superoxide (O_2_•^−^) and hydroxyl radical (•OH), which can cause chromosomal damage associated with its cytotoxic action [41]. In this perspective, we attempted to monitor the intracellular ROS generation in HL-60/MX2 cells by using the fluorogenic ROS probe CM-H_2_DCFDA [42]. The cells preloaded with CM-H_2_DCFDA displayed intense green fluorescence upon treatment of free DNR for 1 h relative to untreated cells (Figure 7a), validating that the increased fluorescence was caused specifically by the internalized DNR, not by oxidative endogenous enzymes in the cells [43]. Notably, we detected dramatically elevated ROS levels in the cells treated with Dauno-MNC, as compared to the free DNR and HA/DNR complex. This finding was further supported by the fluorescence intensity data, in which Dauno-MNC treatment caused a substantial rise in the intracellular ROS levels to ~450%, while only a modest increase in ROS levels (~130%) was induced by the free DNR and HA/DNR complex (Figure 7b). Considering that elevation of intracellular ROS levels has been documented in EGCG-treated leukemia cells [14,15,16], the combinatorial redox activities of DNR and EGCG might be accountable for the enhanced ROS-generating capability of Dauno-MNC. 

Multiple studies have documented that excessive ROS production can cause aberrant DNA damage and mitochondrial dysfunction, ultimately leading to the initiation of apoptotic cell death processes [44,45,46]. The caspase-Glo reagent, a luminogenic caspase substrate, was used to detect the activation of caspases 3/7, which are key effector enzymes involved in the apoptotic process [47]. As presented in Figure 7c, the caspase-3/7 activity of HL-60/MX2 cells was markedly increased to ~334% after treatment of free DNR for 1 day, reflecting its ability to trigger caspase activation. It was noteworthy that Dauno-MNC produced a far greater caspase-3/7 activity (~600%) in HL-60/MX2 cells when compared to the free DNR and HA/DNR complex. This finding was consistent with the intracellular ROS measurements, suggesting that the oxidative stress caused by Dauno-MNC treatment probably contributed to the activation of caspase-dependent apoptosis pathways. Taken together, the above results revealed that Dauno-MNC effectively counteracted the chemoresistance in HL-60/MX2 cells by facilitating the nuclear localization of DNR as well as by elevating intracellular ROS production and caspase-3/7 activities.

To date, there have been only a few studies on the effect of the DNR-EGCG combination as a chemotherapeutic agent. Kitagawa et al. reported that EGCG interfered with the function of the drug efflux transporter P-glycoprotein in multidrug-resistant human epidermal carcinoma KB-C2 cells [48]. Tóth et al. described the ability of EGCG to sensitize THP-1 leukemic cells to DNR by inducing the activation of tumor-suppressor proteins, such as retinoblastoma protein (pRb) and merlin [49]. The EGCG derivative Y6 was found to synergistically augment the efficacy of DNR against human hepatocellular carcinoma by inhibiting the expression of carbonyl reductase 1 (CBR1), an endogenous enzyme involved in the metabolism of anthracycline drugs [50]. Based on our finding combined with the literature, we speculate that multiple biological mechanisms would have probably contributed to the observed chemosensitizing activity of Dauno-MNC. However, we were unable to determine the exact mechanism of action of Dauno-MNC using the current data, and this is an area for future study. Moreover, further studies are warranted to assess the effectiveness and safety of Dauno-MNC in clinically relevant animal models of AML. 

## 3. Materials and Methods

### 3.1. Materials

HA (*M*_w_ = 20 kDa) was purchased from Lifecore Biomedical (Chaska, MN, USA). EGCG was purchased from DSM Nutritional Products Ltd (Heerlen, The Netherlands). Daunorubicin hydrochloride (DNR) was obtained from AbMole BioScience (Houston, TX, USA). Amicon Ultra-15 centrifugal filters were purchased from Merck Millipore Corporation (Darmstadt, Germany). CellTiter-Glo cell viability assay reagent and Caspase-Glo 3/7 assay kit were purchased from Promega (Madison, WI, USA). 5-(and-6)-chloromethyl-2′,7′-dichlorodihydrofluorescein diacetate acetyl ester (CM-H_2_DCFDA) was obtained from Invitrogen (Carlsbad, CA, USA).

### 3.2. Preparation of Dauno-MNC and HA/DNR Complex

HA–EGCG conjugate was synthesized by conjugating EGCG to thiol-end modified HA according to a previous report [17]. An aqueous solution of HA–EGCG conjugate (final concentration: 2–8 mg mL^−1^) was mixed with DNR solution (final concentration: 0.3–0.6 mg mL^−1^) and then incubated for 2 d at 37 °C in an orbital shaker at 50 rpm in a dark place. The resulting nanocomplexes were retrieved by centrifugation for 4 min at 2000× *g* at 25 °C using Amicon Ultra-15 centrifugal filters (*M*_w_ cutoff of 50 kDa) and then purified by repeating dispersion in deionized water and centrifugation three times. The purified nanoparticles were resuspended in 1.5 mL of deionized water and stored at 4 °C until use. For comparison, HA/DNR complex was produced under the same condition except the use of unmodified HA instead of HA–EGCG conjugate. The concentration of HA and DNR used for HA/DNR complex formation was 8 and 0.4 mg mL^−1^, respectively. 

### 3.3. Characterization of Dauno-MNC

The hydrodynamic diameter and zeta potential of Dauno-MNC were characterized using the Zetasizer Ultra Red (Malvern Instruments, Malvern, UK). The stability of Dauno-MNC and HA/DNR complex was evaluated by monitoring the derived count rate over 4 d in 10 mM phosphate-buffered saline (PBS, pH 7.4) at 37 °C. The drug loading efficiency was determined by comparing the absorbance of nanocomplex samples at 480 nm with those of a series of DNR standard solutions (0.2–10 µg mL^−1^). The weight of lyophilized samples was also examined to determine the drug-loading content. 

### 3.4. Fluorescence Spectroscopy

The intermolecular binding between EGCG and DNR was evaluated by fluorescence spectroscopy. Briefly, 1 mL of DNR solution (final concentration: 5 pM) was mixed with 1 mL of EGCG or HA–EGCG conjugate at various final concentrations (0.1–100 pM). After the mixture was transferred into a quartz cuvette, the emission spectra of DNR were acquired at an excitation wavelength at 480 nm on the Cary Eclipse fluorescence spectrometer (Agilent, Santa Clara, CA, USA), with the use of excitation and emission slits of 5 and 5 nm, respectively. For comparison, varying concentrations of unmodified HA were mixed with DNR solution (final concentration: 5 pM) and then examined using the fluorescence spectrometer. 

### 3.5. Drug Release Study

Dauno-MNC, HA/DNR complex or free DNR were transferred to Float-A-Lyzer dialysis tubes with a molecular-weight cutoff of 3.5–5 kDa (Spectrum Laboratories, Rancho Dominguez, CA, USA). The amount of DNR per tube was fixed to 20 µg. The dialysis tubes were immersed in 5 mL of 10 mM sodium citrate buffer (pH 5.5) or 10 mM PBS (pH 7.4) and then incubated at 37 °C on an orbital shaker at 50 rpm with Parafilm sealing to minimize water evaporation. At selected time points, 1 mL of the release fraction was transferred to a quartz cuvette and its absorbance at 480 nm was measured on a Hitachi U-2810 spectrophotometer. After each measurement, the release fraction was returned back to the dialysis tube. The extent of DNR release was determined by comparing the absorbance of nanocomplex samples at 480 nm with those of a series of DNR standard solutions (0.2–10 µg mL^−1^). 

### 3.6. In Vitro Cytotoxicity Study

The human AML cell lines HL-60 and HL-60/MX2 (ATCC, Manassas, VA, USA) were cultured in RPMI 1640 media supplemented with 10% (*v*/*v*) FBS and 1% (*v*/*v*) penicillin/streptomycin. The cells were seeded on white-walled 96-well plates at a density of 10^4^ cells per well and then incubated in 100 µL of 10% FBS-supplemented RPMI media containing Dauno-MNC, HA/DNR complex, free DNR, HA–EGCG or EGCG at various concentrations. After treatment for 3 days, 100 µL of CellTiter-Glo assay reagent was added to each well of the plates. After incubation for 10 min at 25 °C, cellular luminescence was measured using a Tecan Infinite 200 microplate reader (Tecan Group, Männedorf, Switzerland). Results were expressed as percentages of the luminescence signal of analyzed cells relative to untreated controls. 

### 3.7. Evaluation of Synergism 

To assess the synergism between DNR and HA–EGCG conjugate, the combination index (CI) values were calculated with the CompuSyn software (ComboSyn Inc., Paramus, NJ, USA) using the median-effect equation [36]. Briefly, cytotoxicity data were plotted using the linearized median-effect equation:

log(f_a_/f_u_) = m log(D) − m log (D_m_), (1)

where f_a_ is the fraction of killed cells, f_u_ is the fraction of survived cells, D is the dose applied and D_m_ is the median effective dose. The resultant plot gave the slope of m and the *y*-axis intercept of – m log (D_m_). Based on the mutually exclusive model, CI was determined by an equation:

CI = (D)_1_/(ED_x_)_1_ + (D)_2_/(ED_x_)_2_, (2)

where (ED_x_)_1_ and (ED_x_)_2_ are the doses of single agents (free DNR or HA–EGCG) that produce x% effect, while (D)_1_ and (D)_2_ are the doses of DNR and HA–EGCG needed to produce the same effect in the combination regimen, respectively. The combination was considered synergistic when CI < 1, additive when CI = 1 and antagonistic when CI > 1. 

### 3.8. Flow Cytometry and Fluorescence Microscopy

To examine CD44 expression levels, HL-60 and HL-60/MX2 cells (3 × 10^5^ cells) were stained with FITC-tagged anti-human CD44 antibody (clone BJ18, BioLegend) for 1 h at 4 °C. The cells were rinsed three times with ice-cold PBS containing 0.1% (*w*/*v*) bovine serum albumin prior to flow cytometry analysis by LSRFortessa X20 cell analyzer (BD Biosciences, Franklin Lakes, NJ, USA). To evaluate the extent of cellular uptake, HL-60/MX2 cells were seeded on 6-well plates at a density of 20 × 10^4^ cells per well and then incubated in 2 mL of 10% FBS-supplemented RPMI media containing free DNR, HA/DNR complex or Dauno-MNC without or with excess free HA (10 mg mL^−1^) at a final DNR concentration of 1.6 µM. After 4 h of incubation, the cells were washed with ice-cold PBS three times and then analyzed by LSRFortessa X20 cell analyzer (BD Biosciences). For fluorescence microscopy, 20 × 10^5^ cells were firstly cytospun onto glass slides using Cytospin 4 Cytocentrifuge (Thermo Scientific). Cells were then fixed with 4% paraformaldehyde solution for 15 min prior to washing with PBS. DAPI (4’,6-diamidino-2-phenylindole, Sigma-Aldrich D8417, St. Louis, MI, USA) was added to stain nucleus of cells. All images were captured by the ZEISS Axio Scan.Z1 and processed using the Zen lite software. 

### 3.9. Detection of Intracellular ROS Generation 

Intracellular ROS generation was evaluated by using the fluorogenic ROS probe CM-H_2_DCFDA [42]. Briefly, HL-60/MX2 cells (1 × 10^6^ cells) were resuspended in 2 mL of pre-warmed PBS containing 10 μM of CM-H_2_DCFDA. After 30 min of dye loading, the cells were rinsed with PBS twice and then incubated in 5 mL of 10% FBS-supplemented RPMI media for additional 30 min to allow for hydrolysis of the acetate groups of the probe by cellular esterases. The cells were seeded on black-walled 96-well plates at a density of 2 × 10^4^ cells per well and then incubated in 100 µL of 10% FBS-supplemented RPMI media containing Dauno-MNC, HA/DNR complex or free DNR at a final DNR concentration of 1.6 µM. After 1 h of incubation, cellular fluorescence was measured on a Tecan Infinite 200 microplate reader (Tecan Group, Switzerland) at an excitation and emission wavelength of 485 and 525 nm, respectively. The fluorescence images were acquired on Olympus IX83 inverted microscope and processed using cellSens software (Olympus Corporation, Tokyo, Japan). 

### 3.10. Assessment of Caspase-3/7 Activity 

Induction of apoptosis was assessed by measuring caspase-3/7 activity using Caspase-Glo 3/7 assay kit. Briefly, HL-60/MX2 cells were seeded on white-walled 96-well plates at a density of 2 × 10^4^ cells per well and then incubated in 100 µL of 10% FBS-supplemented RPMI media containing Dauno-MNC, HA/DNR complex or free DNR at a final DNR concentration of 1.6 µM. After 1 d of incubation, 100 µL of Caspase-Glo 3/7 assay reagent was added to each well of the plates and then incubated for 1 h at 25 °C in a dark place. The cellular luminescence was measured using a Tecan Infinite 200 microplate reader (Tecan Group, Mennedoff, Switzerland). Results were expressed as percentages of the luminescence signal of analyzed cells relative to untreated controls.

### 3.11. Statistical Analysis

All data are presented as mean ± standard deviation (SD). Statistical analysis was conducted using the OriginPro 9 software (one-way ANOVA with Tukey’s post hoc multiple comparison test). Significance was determined at *p* values smaller than 0.05.

## 4. Conclusions

In the present study, a self-assembled micellar nanocomplex composed of a DNR and HA–EGCG conjugate was developed, for the first time, to achieve synergistic reversal of chemoresistance in leukemia. The nanocomplex, termed Dauno-MNC, exhibited high stability at extracellular fluid pH, but rapidly released the encapsulated DNR upon exposure to acidic endosomal pH. More importantly, Dauno-MNC greatly amplified the therapeutic efficacy of DNR on multidrug-resistant HL-60/MX2 cells in a synergistic manner. A series of cell experiments demonstrated that the chemosensitizing effect of Dauno-MNC was mediated by an elevation of nuclear accumulation, intracellular ROS production and caspase-3/7 activities. This work provides new insights into the design of synergistic nanomedicine overcoming chemoresistance for more effective leukemia therapy.

## Data Availability

Not applicable.

## Supplementary Materials

The following supporting information can be downloaded at: https://www.mdpi.com/article/10.3390/ijms24010381/s1, Figure S1: Representative photograph of Dauno-MNC and HA/DNR complex; Figure S2: Fluorescence emission spectra (λ_ex_ = 480 nm) of DNR solution (5 pM) mixed with various concentrations of HA; Figure S3: Cytotoxicity of Dauno-MNC, HA–EGCG and EGCG against HL-60/MX2 cells as a function of EGCG unit concentration; Figure S4: Flow cytometric detection of CD44 in HL-60 and HL-60/MX2 cells labeled without or with FITC-tagged anti-CD44 antibody.
