# Supplementary material for: Self-Assembled Daunorubicin/Epigallocatechin Gallate Nanocomplex for Synergistic Reversal of Chemoresistance in Leukemia
Source: Int J Mol Sci. 2022 Dec 26;24(1):381. doi: 10.3390/ijms24010381 (PMC9820275; doi:10.3390/ijms24010381)
Supplement: Supplementary file 1 [file ijms-24-00381-s001.zip › ijms-2092876-supplementary.pdf]

Supporting Information for

Self-Assembled Daunorubicin/Epigallocatechin Gallate Nanocomplex for Synergistic Reversal of Drug Resistance in Leukemia

Ki Hyun Bae [1], Fritz Lai [2], Betul Oruc [1], Motomi Osato [3], Qingfeng Chen [2], and Motoichi Kurisawa [1,4,*]

[1]Institute of Bioengineering and Bioimaging, 31 Biopolis Way, The Nanos, Singapore 138669; khbae@ibb.a-star.edu.sg (K.H.B.); betulaltin@alumni.sabanciuniv.edu (B.O.)

[2]Institute of Molecular and Cell Biology, 61 Biopolis Drive, The Proteos, Singapore 138673; sclai@imcb.a-star.edu.sg (F.L.); qchen@imcb.a-star.edu.sg (Q.C.)

[3]Cancer Science Institute of Singapore, National University of Singapore, 14 Medical Drive, Singapore 117599.; csimo@nus.edu.sg (M.O.)

[4]School of Materials Science, Japan Advanced Institute of Science and Technology, 1-1 Asahidai, Nomi, Ishikawa 923-1292, Japan

*Correspondence: kurisawa@jaist.ac.jp

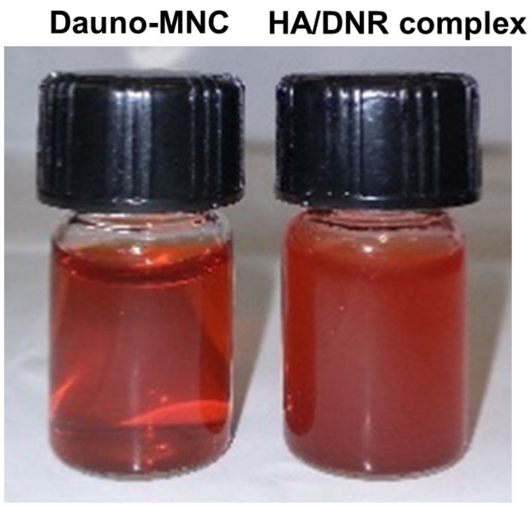

Figure S1. Representative photograph of Dauno-MNC (left) and HA/DNR complex (right).

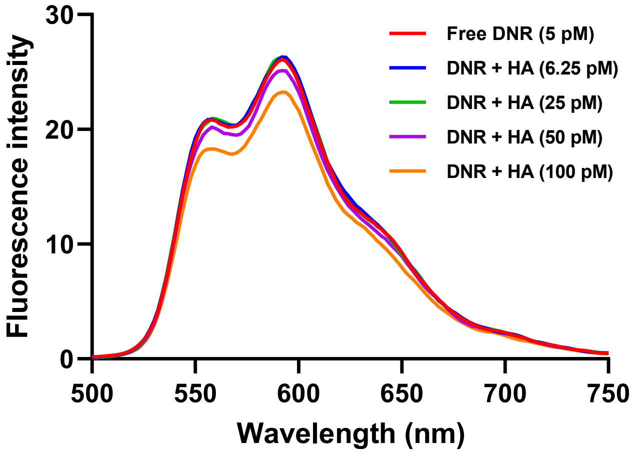

Figure S2. Fluorescence emission spectra (λ_{ex}=480 nm) of DNR solution (5 pM) mixed with various concentrations of HA.

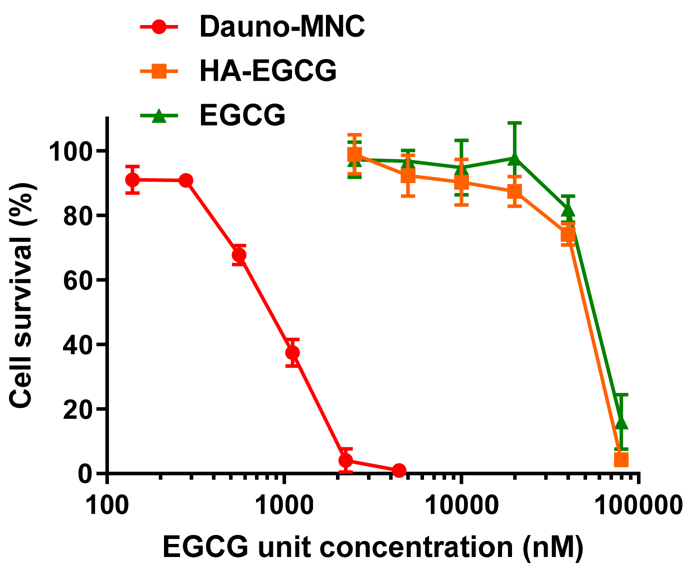

Figure S3. Cytotoxicity of Dauno-MNC, HA-EGCG and EGCG against HL-60/MX2 cells as a function of EGCG unit concentration. Mean ± SD (n = 4).

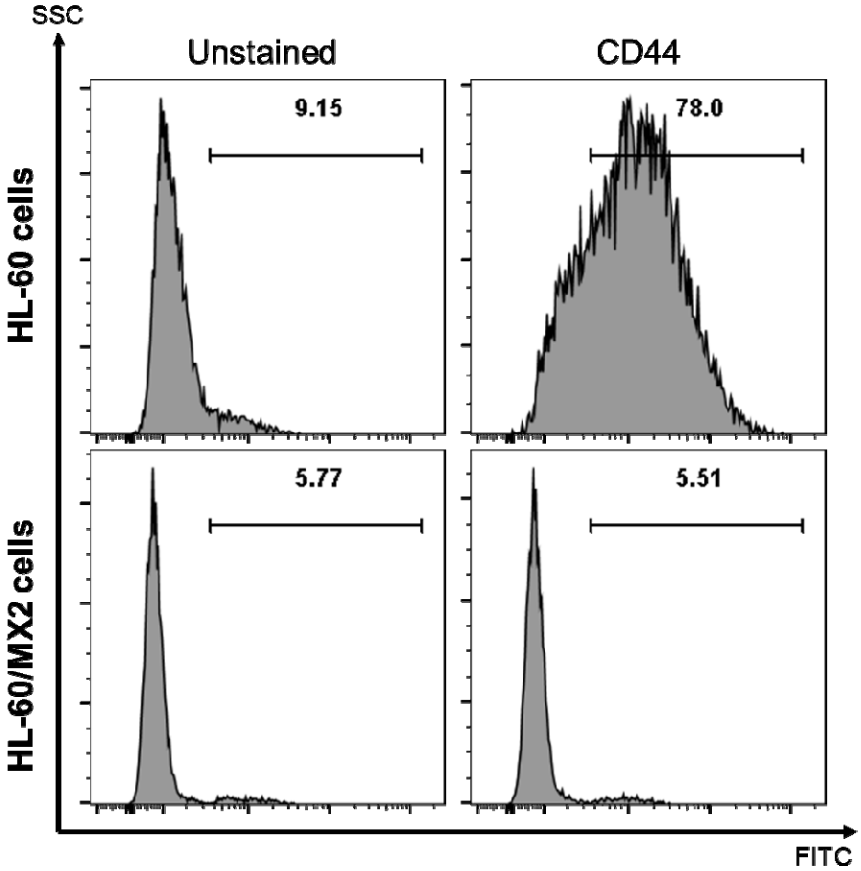

Figure S4. Flow cytometric detection of CD44 in HL-60 and HL-60/MX2 cells labeled without (left panel) or with FITC-tagged anti-CD44 antibody (right panel).
